# Altering the Mechanical Load Environment During Growth Does Not Affect Adult Achilles Tendon Properties in an Avian Bipedal Model

**DOI:** 10.3389/fbioe.2020.00994

**Published:** 2020-09-02

**Authors:** Kavya Katugam, Suzanne M. Cox, Matthew Q. Salzano, Adam De Boef, Michael W. Hast, Thomas Neuberger, Timothy M. Ryan, Stephen J. Piazza, Jonas Rubenson

**Affiliations:** ^1^Biomechanics Laboratory, Department of Kinesiology, The Pennsylvania State University, University Park, PA, United States; ^2^Integrative and Biomedical Physiology, The Pennsylvania State University, University Park, PA, United States; ^3^Huck Institutes of the Life Sciences, The Pennsylvania State University, University Park, PA, United States; ^4^Biedermann Lab for Orthopaedic Research, Perelman School of Medicine, University of Pennsylvania, Philadelphia, PA, United States; ^5^Department of Biomedical Engineering, The Pennsylvania State University, University Park, PA, United States; ^6^Department of Anthropology, The Pennsylvania State University, University Park, PA, United States

**Keywords:** tendon, growth, stiffness, modulus, development

## Abstract

Tendon mechanical properties respond to altered load in adults, but how load history during growth affects adult tendon properties remains unclear. To address this question, we adopted an avian model in which we altered the mechanical load environment across the growth span. Animals were divided at 2 weeks of age into three groups: (1) an exercise control group given the opportunity to perform high-acceleration movements (EXE, *n* = 8); (2) a sedentary group restricted from high-intensity exercise (RES, *n* = 8); and (3) a sedentary group also restricted from high-intensity exercise and in which the gastrocnemius muscles were partially paralyzed using repeated bouts of botulinum toxin-A injections (RES-BTX, *n* = 8). Video analysis of bird movement confirmed the restrictions eliminated high-intensity exercise and did not alter time spent walking and sitting between groups. At skeletal maturity (33–35 weeks) animals were sacrificed for analysis, consisting of high-field MRI and material load testing, of both the entire free Achilles tendon and the tendon at the bone-tendon junction. Free tendon stiffness, modulus, and hysteresis were unaffected by variation in load environment. Further, the bone-tendon junction cross-sectional area, stress, and strain were also unaffected by variations in load environment. These results suggest that: (a) a baseline level of low-intensity activity (standing and walking) may be sufficient to maintain tendon growth; and (b) if this lower threshold of tendon load is met, non-mechanical mediated tendon growth may override the load-induced mechanotransduction signal attributed to tendon remodeling in adults of the same species. These results are important for understanding of musculoskeletal function and tendon health in growing individuals.

## Introduction

Tendon mechanical properties change in response to variations in mechanical load history. In adult animals, including humans, increased loading increases both tendon stiffness and modulus, whereas decreased loading has had the opposite effect (for reviews see: Wren et al., 2000; Wang, 2006; Magnusson et al., 2008; Bohm et al., 2015).

While a causal link between load stimulus and tendon properties has been demonstrated for adult tissue, the few studies that have examined how variation in load across the growth period affect tendon material properties have produced contradictory findings. A comparison of adolescent athletes to non-athletes provided indirect evidence that long-term training during growth may result in increased patellar tendon stiffness (Mersmann et al., 2017a; Charcharis et al., 2019). A similar comparison between pre-adolescent athletes and non-athletes, however, found no differences in Achilles tendon stiffness (Pentidis et al., 2019). Short-term (10-week) resistance training interventions in both typically developing pre-adolescent children (Waugh et al., 2014) and in children with cerebral palsy (Kalkman et al., 2019) have been shown to increase Achilles tendon stiffness. We are not aware of any longer-term intervention studies of tendon adaptation in human children, but a 1-year longitudinal study tracking adolescent volleyball athletes undergoing strenuous training and minimally active control subjects found no significant between-group differences in the changes in patellar tendon stiffness (Mersmann et al., 2016). In this study, and in others by these authors (for a review, see Mersmann et al., 2017b), it is proposed that tendon has a slower and less pronounced response to altered load during growth compared to muscle, a phenomenon that may lead to an imbalance in the development of muscle and tendon strength.

Non-human animal models may provide insight into load-induced tendon plasticity across longer growth periods. For example, domestic fowl reared in cages that restrict movement developed Achilles tendons with a lower stiffness and elastic modulus compared to the same species reared in large pens that allowed for walking and running (Nakagaki et al., 2007), although detailed descriptions of activity in their groups were not included. More severe disuse models have found inconsistent results. Walsh et al. (1993) reported a reduction in stiffness and strength in rabbit knee ligament after immobilization. On the other hand, using botulinum toxin to paralyze murine muscle has resulted in tendon stiffness that decreases (Schwartz et al., 2013), increases (Khayyeri et al., 2017) or remains unaltered (Eliasson et al., 2007) when these interventions are applied during growth. Both of the latter two studies report a reduction in hysteresis in the unloaded tendon, which the authors interpret as impaired tendon damping (Eliasson et al., 2007; Khayyeri et al., 2017). An absence of altered tendon stiffness and tenocyte histology has similarly been observed in equine studies implementing trotting and galloping training across a substantial portion of the animals’ growth span (Kasashima et al., 2008; Stanley et al., 2008). Likewise, Achilles tendon size and collagen content were found to be unaltered by high-intensity running training in growing domestic fowl (Curwin et al., 1988). Inconsistencies in the literature, such as those above, make it difficult predict how altered load during childhood will affect adult tendon. Overcoming this shortcoming is especially important in light of the trends of inadequate physical activity in growing children (Guthold et al., 2020).

Some of the uncertainty in the influence of load on tendon properties arises due to differences in measurement technique and mode, magnitude and duration of altered loads in the previous studies. For example, uncertainty in the load thresholds required for initiating tendon adaptation (Lavagnino and Arnoczky, 2005; Arampatzis et al., 2007) and their sensitivity to load frequency (Arampatzis et al., 2010) make it difficult to interpret the various outcomes from experimental load interventions. Our understanding of load-induced tendon adaptations during growth is hindered further because a number of prior investigations have not assessed tendon mechanical properties in functional contexts. In these studies, tendon properties have been assessed from portions of tendon, and then assumed to represent the entire tendon. The distal part of tendons, including the tendon-bone junction, are common sites of rupture under load (Jack, 1950; Woo et al., 1983; Woo and Buckwalter, 1988; Jarvinen, 1992), very likely due to their greater strain and stress, and smaller cross sectional area (Woo et al., 1983; Wren et al., 2000, 2001, 2003). Therefore, testing methods that exclude distal tendon regions and the bone-tendon junction may result in an over- or under-estimation of the whole-tendon mechanical properties and obscure their relevance to locomotor function.

The aim of this study was to address these limitations by (1) amplifying any possible load-induced changes in tendon properties by examining both a range of altered tendon loading, and by altering load across the entire growth period; and (2) by quantifying both whole tendon and regional properties relevant to *in vivo* function. To accomplish this, we applied a chronic load-reduction approach to the Achilles tendon in an avian bipedal model (guinea fowl; *Numida meleagris*). This permits alteration of the load stimulus over a large range of tissue size and over a substantially greater portion of the animals’ growth span compared to most other model systems. Guinea fowl have substantially greater body-mass growth compared to rodent models (two orders of magnitude increase in body mass) and juvenile guinea fowl can locomote as early as 1-day-old. In this model system, we designed the experiment to induce two levels of load reduction to compare against controls. First, we reduced tendon load by restricting locomotor behavior in one group. This was achieved by eliminating high-intensity activity via space restriction (Cox et al., 2020). Secondly, in another group, we aimed to generate a more pronounced Achilles tendon load reduction by eliminating high-intensity movements and further paralyzing the gastrocnemius muscles using botulinum toxin-A (BTX-A), a neurotoxin known to offload muscle and tendon (Longino et al., 2005; Schwartz et al., 2013). In all groups we tracked the activity level throughout maturation. The experimental design intended to induce levels of decreased load over a much longer duration than most previous studies and to be able to relate these results to changes in activity level.

We assessed tendon properties of birds reared in these conditions in two functional contexts post-growth. First, we investigated the spring-like quality of the tendon by measuring the stiffness, modulus and hysteresis of the entire free tendon. The properties of the full intact tendon most directly reflect its overall capacity to store and release elastic energy during locomotion, as well as how it will influence muscle fiber mechanics and energetics (Lichtwark and Wilson, 2008; Roberts and Azizi, 2011; Konow et al., 2012). Second, we assessed tendon properties at the bone-tendon junction. Similar to the human Achilles, the bone-tendon junction is the tendon region with the smallest cross sectional area in guinea fowl and is thus important for understanding tendon strength. Based on the response known to occur in adult tendon, we hypothesized that tendon would adapt in a load-dependent manner. Specifically, we predicted that the overall free Achilles tendon stiffness, modulus, and hysteresis would be reduced in animals in which high-intensity movements were eliminated across the growth period, and further reduced in animals administered (BTX-A). Additionally, we predicted that the distal tendon at the bone-tendon junction would exhibit smaller cross-sectional areas and undergo larger stress and strain during loading.

## Materials and Methods

### Animals

Twenty-four one-day old guinea fowl keets were obtained from a regional breeder (Guinea Farm; New Vienna, IA, United States). At 2-weeks of age, animals were divided into three groups: an exercise control group (EXE, *n* = 8; 3 female, 5 male), a restricted movement group (RES, *n* = 8; 3 female, 5 male), and a Botox group (RES-BTX, *n* = 8; 3 female, 5 male). Animals were pen-raised (see below for details) in a 12-h/12-h light/dark cycle, with both food and water provided *ad libitum*. The experimental design was approved by The Pennsylvania State University Institutional Animal Care and Use Committee (IACUC; protocol #46435) and the Institutional Biosafety Committee (IBC; protocol #47306).

### Movement Analysis

Video recordings (Foscam; C2 1080p HD cameras; Houston, TX, United States) of each pen were acquired to quantify the daily movement patterns of the animals. A single camera was placed over each pen, with each camera’s field-of-view capturing the group pen. Videos were recorded four times per day, across the growth period. A random subset of 60 videos (20 EXE, 20 RES, 20 RES-BTX) recorded during the 12-h light cycle were analyzed using methods outlined previously by Cox et al. (2020). Briefly, the first 5 min of each selected video was analyzed with a custom-written MATLAB script (The MathWorks, Natick, MA, United States) that allowed users to timestamp observed activities (i.e., walking, standing, sitting, and high-acceleration actions including jumps and sprint actions) via keystroke. The percent time spent walking, standing and sitting was assessed as group ensemble, reflecting the activity of the pen, while high-acceleration movements were recorded on an individual animal basis. Each individual video was analyzed three times and averaged. These were used to compute group averages for comparisons. Individual birds were not identifiable in any videos, so bird-specific analyses were not conducted.

### Treatment

All RES and RES-BTX animals were group-reared in restricted-movement pens (1 m^2^ at maturity) without perches, eliminating the ability for birds to jump and highly restricting running and sprinting (Cox et al., 2020). All EXE animals were group-reared in larger pens (3.14 m^2^) with perches, in which animals were free to perform high-acceleration movements expected to result in high tendon loads, including running, sprinting and jumping to and landing from perches.

We chose to examine the Achilles tendon in this study, in part because of its prominent functional role in movement (Daley and Biewener, 2003; Marsh et al., 2004; Henry et al., 2005), and because it is in series with large muscles (gastrocnemius) that are readily treated with Botulinum toxin injections. BTX-A (Allergan, Irvine, CA, United States) was applied to the lateral and medial gastrocnemius muscles (LG and MG) to induce a local unloading effect on the Achilles tendon that would be greater than that achieved by restricting movement alone. Starting at 7–8 weeks of age, RES-BTX animals received bilateral BTX-A injections (4 units (LD50)/kg per leg) into the LG and MG muscles while under general anesthesia (1.5% isoflurane). BTX-A was combined with 0.9% sodium chloride to make a concentration of 10 units/ml. BTX-A injections were administered at multiple locations in each muscle, covering the proximal-distal and medio-lateral muscle regions. Previous studies in rabbits have indicated that an amount of 3.5 units/kg was sufficient to elicit a functional muscle impairment (Longino et al., 2005; Fortuna et al., 2015).

At 7–8 weeks of age, identifying muscle boundaries from palpation alone was difficult, so the first BTX-A administration was performed using a sterile dermatotomy procedure. Skin incisions were closed with absorbable suture (4.0 Monocryl). All subsequent BTX-A injections were administered via percutaneous injection. BTX-A was re-administered every 5 weeks (week 12–13; week 17–18; week 22–23) for a total of four injections. After each BTX-A administration, RES-BTX animals were monitored for 1–2 days in 0.6 m^2^ individual cages before returning to group-housing. The RES and EXE group animals received a sham saline injection in the gastrocnemius muscles using the same volume injection and at the same frequency. At 33–35 weeks of age, animals were weighed and euthanized (pentobarbital >1.6 mg/kg), at which time animals had reached sexual and skeletal maturity.

### Tendon Imaging

**FIGURE 1:**
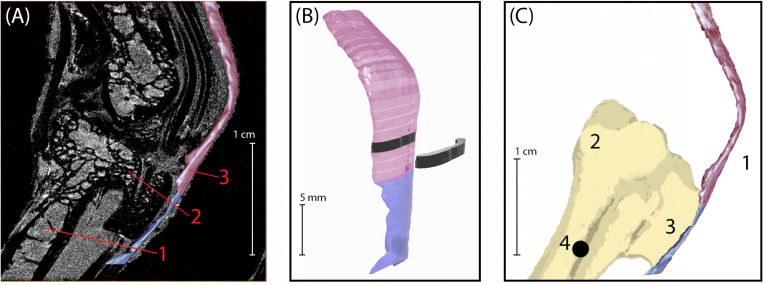
**(A)** Sagittal plane MRI of the guinea fowl ankle joint. 1: tarsometatarsus bone (TMT). 2: Hypotarsus of the TMT, where the Achilles tendon inserts. 3: Achilles Tendon. **(B)** 3D rendering of the Achilles tendon, showing segmentation used to calculate cross-sectional area. The transition between colors indicates the bone-tendon-junction, which corresponds to the three-dot horizontal marker painted onto the tendons for video analysis (see Figure 2). Each tendon slice (shown in black) is 1 mm in thickness. **(C)** Medial view of bone-tendon specimen prepared for material testing. Prior to testing, superficial connective tissue was removed to expose the Achilles tendon (1), which inserts on the proximal end of the tarsometatarsus (2) at the hypotarsus (3). A 1.2 mm diameter hole (4) was drilled into the distal end of the tarsometatarsus bone, proximal to the hypotarsus, to allow for loading and clamping of specimen into material testing rig.

Following euthanasia, the pelvic limb of each animal was split along the midline of the pelvis. The right limb was kept fresh-frozen (−20°C) for tendon analysis. Tendon volume and cross-sectional area (CSA) were assessed with high-field MRI (7T Biospec Avance III HD; Bruker Biospin, Billerica, MA, United States; Figure 1A). Before MR imaging, limbs were placed onto an acrylic board. Using wooden dowels and zip ties to mount the limbs allowed for similar orientation for all animals. The small diameter of the MRI bore required the limb to be positioned in an extended posture. By keeping the knee extended a small amount of tension was maintained on the tendon keeping the tendon alignment similar between specimens. Once mounted, limbs were sprayed with saline and covered in plastic wrap to retain moisture during the scanning procedure. After preparation, limbs were inserted into a 60 mm inner diameter quadrature driven birdcage resonator and placed into the isocenter of the magnet. A standard three-dimensional gradient echo imaging sequence with fat suppression yielded a 100-micron isotropic resolution within 33 min (repetition time: 40 ms; echo time: 3.9 ms; field of view (FOV): 35 mm × 25 mm × 20 mm; matrix size: 350 × 250 × 200; averages: 1). Data were zero filled by a factor of two in each direction using a custom MATLAB script resulting in a 50-micron isotropic pixel resolution. Image segmentation was performed using the lasso tool in Avizo (Thermo Fisher Scientific; Waltham, MA, United States).

Once segmented, tendon files were exported as stereolithography (STL) files that were imported into Rhinoceros 3D modeling software (Robert McNeel & Associates; Seattle, WA, United States) for determining overall and regional cross-sectional areas. Meshes were converted to polysurfaces and sliced every millimeter perpendicular to the lateral surface of tendon (Figure 1B). The cross-sectional area for each slice surface was calculated using the area tool in Rhinoceros 3D and recorded.

### Tendon Preparation

Following MR imaging, the gastrocnemius muscles were detached from their origins on the tibiotarsus and femur. Muscle tissue was carefully dissected away from the proximal tendon aponeurosis. The Achilles tendon remained intact and attached to the hypotarsus, the attachment site for the Achilles tendon on the tarsometatarsus (TMT).

**FIGURE 2:**
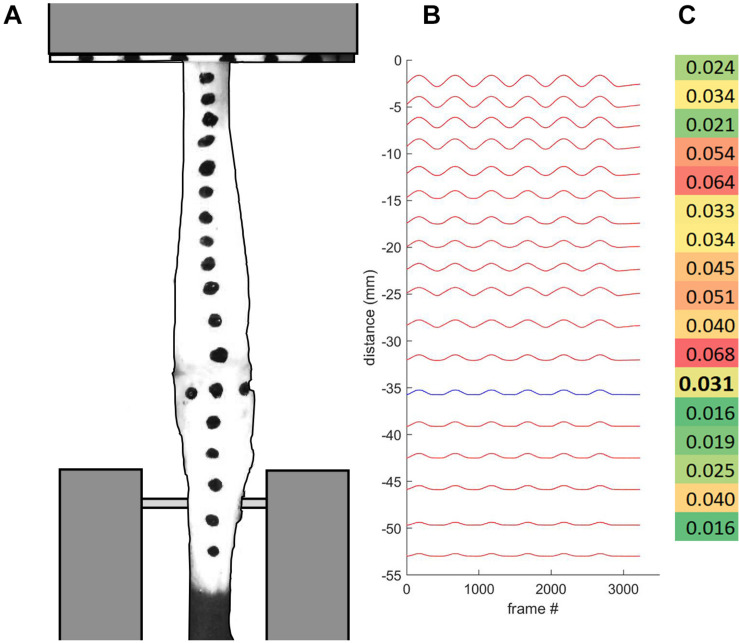
**(A)** Tendon specimen were marked for regional strain analysis, and loaded into custom-built clamps. The bone-tendon junction is indicated with three horizontally aligned marks. **(B)** Displacement plot indicates bone-tendon junction distal region in blue. **(C)** Strain profile map indicates higher regional strain values in red and lower regional strain values in green. Bolded value corresponds with the tendon region proximal to the bone-tendon junction.

The fascia around the posterior ankle was carefully removed so as to isolate the free Achilles tendon. A 1.2 mm diameter hole was drilled in the proximal end of the TMT to secure the bone during material testing (Figure 1C). To facilitate regional strain analysis along the free tendon, small marks were placed along the length of the free tendon with insoluble acrylic lacquer paint (Krylon Products Group, Cleveland, OH, United States). The space between each mark was approximately 5% of the TMT length for each given sample (Figure 2A), resulting in between 13 and 20 marks per tendon. The TMT was used to standardize the tendon marks, as opposed to the tendon slack length, because the later was established after mounting the tendon (see below). The bone-tendon junction was indicated using a horizontal row of three marks, perpendicular to the length of the tendon (Figure 2A). Video recordings (Grasshopper3 GS3-U3-23S6C; FLIR Systems, Inc., Wilsonville, OR, United States) were used to track the tendon strain through marker movement. The entire limb was kept hydrated using a saline bath for a minimum of 30 min prior to material testing and throughout the testing procedure.

### Tendon Material Testing

**FIGURE 3:**
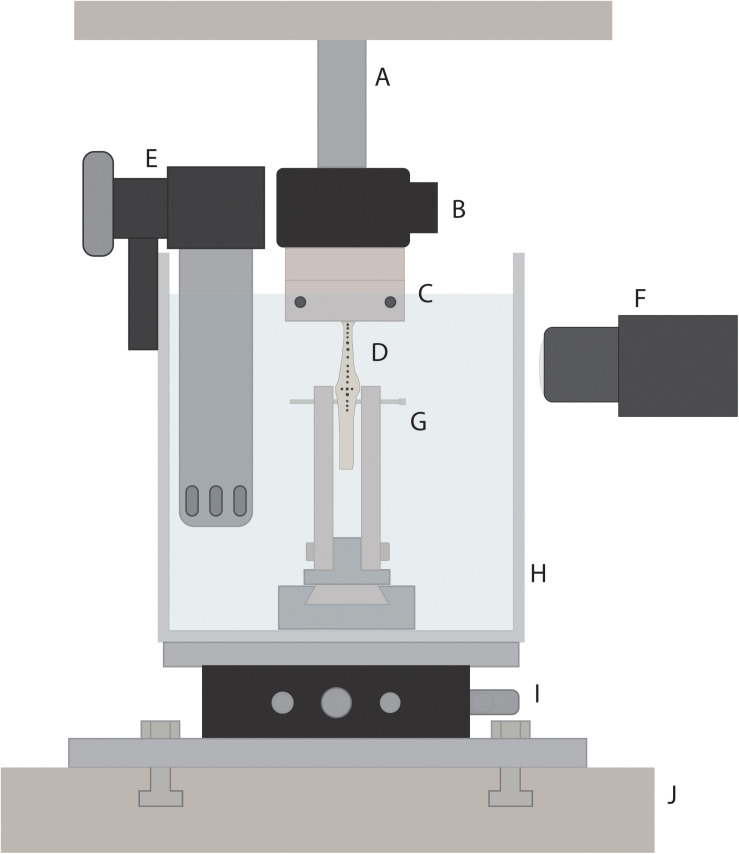
Schematic of custom-built rig for tendon material testing. The rig was inserted into a material testing system (J; MTS Systems Corporation, Minneapolis, MN, United States) to allow for loading of tendon samples. Tendon specimen (D) were loaded via a 3D-printed tendon tissue clamp (C) at the proximal end, and a bone mount (G) at the distal end. A 50-pound load cell (B) connected the proximal end of the tendon specimen to the MTS linear actuator (A). An xy-stage (I) was used to orient the tendon, allowing for load to be applied along the long axis of the tendon. Tendons were submerged in a saline bath (H) that was heated to the average active body temperature for guinea fowl (41.5°C) using a water heater (E). A high speed camera (F) was used to track tendon marks during loading.

Mechanical properties of the tendons were measured via a material testing system (858 Mini Bionix II; MTS Systems Corp; Eden Prairie, MN, United States) using a custom-built rig (Figure 3). Samples were mounted vertically via custom clamps on the tendon aponeurosis and the TMT, and attached to a 50-pound load cell (MTS Systems Corp; Eden Prairie, MN, United States). The upper clamp gripped the entire aponeurosis of each sample, leaving only the free tendon exposed to loading. The transition from the free tendon to aponeurotic tendon was clearly identifiable by a marked change in tendon tissue thickness. The sample was clamped within a saline bath kept at the average active body temperature for guinea fowl (41.5 degrees Celsius; Prinzinger et al., 1991).

A tensile testing protocol was adapted from a previous study on explanted tendon (Miller et al., 2012). After tendons were mounted into the custom-built rig, the upper clamp was lowered until the free-tendon was visibly slack, after which the force reading was zeroed. The upper clamp was then raised, elongating the tendon, until the force reading was approximately 5 N, and then was lowered again until the force first read 0.000 ± 0.001 N. The displacement reading was again zeroed and the free tendon slack length for each sample was measured using digital calipers as the distance from the middle of the hypotarsus to the start of the aponeurotic tendon, corresponding to the bottom of the upper clamp (Figure 3).

Tendons were first preconditioned by cyclic loading from 0 to 0.005 strain at 0.1 Hz for 10 cycles following Schmidt et al. (2019). Strain (ε) was calculated as the clamp displacement (ΔL) divided by the free tendon slack length (*L*_*0*_, mm):

$$\varepsilon =\frac{\Delta \,L}{L_{0}}.$$

After preconditioning, tendons were loaded cyclically from 0 to 0.05 strain at 0.1 Hz for 20 cycles. Force and displacement were sampled at 102.4 Hz. Video recordings of the last five loading cycles for each tendon were captured at 50 Hz. Each sample video was spatially calibrated using still images of the clamped tendon, with a mm scale bar positioned in line with the tendon. Three images were taken of each sample, and pixel-to-millimeter conversion factors were computed for each image and averaged.

A custom-built LED circuit was used to synchronize video data with MTS output data. During the first filmed loading cycle, a TTL signal from the MTS triggered the LED light to turn on (5V high). During the last filmed loading cycle, the TTL voltage turned low, triggering the LED light to turn off. The recorded data were synchronized to the video data using a Boolean TTL variable (0–1) stored in the MTS data, the frames of video in which the LED turned on and off, and by time normalizing the MTS and video data. This was achieved by down-sampling the MTS data with spline interpolation.

### Tendon Functional Stiffness, Elastic Modulus, and Hysteresis Analysis

First, force (*F*, N) and displacement (Δ*L*, mm) data were shifted to start at (0,0), as small errors in initial length measurements due to pre-conditioning cycles resulted in shifted force-length curves. As a result, maximum tendon strain exhibited some variability (mean maximum strain was 0.045 ± 0.0024 with a range between 0.04 and 0.048 strain).

**FIGURE 4:**
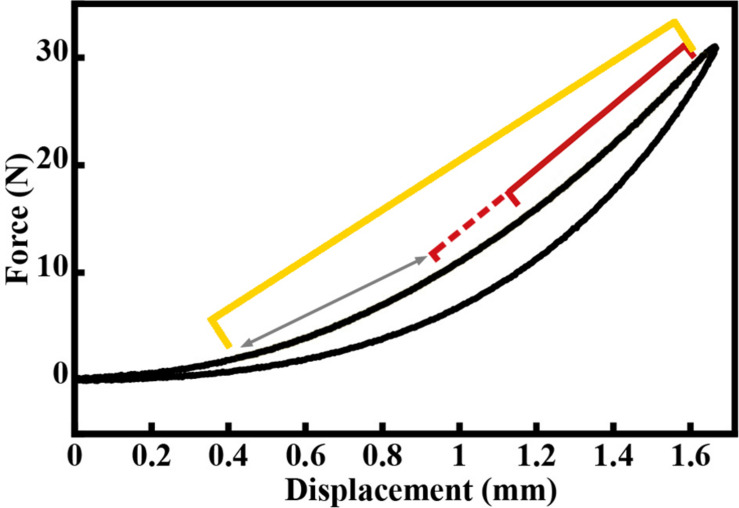
Example force-displacement curve for a load-unload cycle of the Achilles tendon, exhibiting a typical j-shape toe region. Yellow bracket represents 25–95% of loading curve. The red bracket represents the region within the 25–95% range that resulted in the lowest *RMS*_*error*_ of the linear fit, which was then used to compute the stiffness value for this loading cycle. The red dashed bracket and arrow represents other regions for which linear fits were assessed. These regions spanned the entire length of the 25–95% region, always ending at 95% of the loading curve.

Force and displacement data from the last five loading cycles were analyzed for each tendon using custom-written routines in MATLAB. Force data were filtered using a 5 Hz low-pass filter. Force-displacement curves were plotted for each sample. The force-displacement curves exhibited a typical J-shape with a clear toe region (Figure 3). Stiffness was calculated by fitting a line to the force-length curve beyond the toe region, within the range of 25–95% of the tendon’s maximal length (Figure 4). The region within this range that resulted in the lowest *RMS*_*error*_ of the linear fit,

$$R\,M\,S_{\text{error}}=\sqrt{\frac{\sum {\left(Y_{\text{fit}}-Y_{\text{exp}}\right)}^{2}}{\mathrm{number}\,\mathrm{points}}},$$

was used to compute stiffness, with the minimum region set to 15% of the total tendon length and ending at 95% of the maximum tendon length (Figure 4). The slope of this linear fit region was used as the cycle stiffness. This process was repeated for each of the last five loading cycles for each sample, and these stiffness values were averaged across cycles to determine the functional tendon stiffness (*K*_*func*_, N/mm) for each sample. The fitting routine was done using a custom function in MATLAB (The MathWorks, Natick, MA, United States).

Stress (σ, N/mm^2^) for each sample were calculated using the average CSA for each tendon (*CSA*_*avg*_, mm^2^). The modulus of elasticity (*E*, MPa) for each sample was computed from the stress-strain curves in the same manner as described for determining *K*_*func*_. To better characterize the tendon mechanical properties, we assessed the association between *K*_*func*_and *E* and between *K*_*func*_ and *CSA*_*avg*_ in each group and across all groups using Pearson product moment correlations.

Finally, we assessed tendon hysteresis, which can influence the ability of the tendon to return energy during cyclical movement (Bennett et al., 1986; Shadwick, 1990). Strain energy was calculated as the area under the entire extension portion of each of the last five stress-strain cycles, including the toe region. The average energy storage (ϕ_in_) was computed from these five cycles for each sample. The average energy recovered from each sample (ϕ_out_) was calculated as the average area under the unloading portion of the last five stress-strain cycles. The energy lost (ϕ_loss_) was calculated as the difference between ϕ_in_ and ϕ_out_ (ϕ_loss_ = ϕ_in_−ϕ_out_). Hysteresis (*H*) was defined for each sample as:

$$H=\frac{\phi_{\text{loss}}}{\phi_{\text{in}}}.$$

### Regional Tendon Analysis

Regional strain at the bone-tendon junction was determined for each tendon sample. The ink marks on the calibrated video of the last five loading cycles for each tendon were auto digitized (ImageJ, MTrack2 Plug-in) to extract their position data. The bone-tendon junction region was defined as the section of the tendon between the center of the hypotarsus (identified by horizontal markers, as seen in Figure 2) and the closest proximal tendon mark. Our standardized marking procedure resulted in this region being between 3 and 5 mm (4.15 and 7.01% of *L*_*0*_). A maximal regional strain value was calculated by dividing the regional displacement by the region length at *L*_*0*_. The maximal regional displacement value used in this computation was taken when the total tendon strain was 0.04, since all tendons were strained at least to this amount. The bone-tendon junction tendon stress was computed as the ratio of the force applied to the tendon at this strain and the minimum cross-sectional area within the most distal tendon region (bone-tendon junction region CSA; *CSA*_*BTJ*_, mm^2^). The *CSA*_*BTJ*_ of each sample was extracted from the MRI data. This was done by matching the bone-tendon junction region on the MR images to the video data. In the video recordings, the bone-tendon junction was identified prior to material testing and marked with a horizontal row of three dots. The bone-tendon junction was identified in MR images by observation since the bone and the tendon were both identifiable. Above the tendon attachment site, the tendon CSA consistently increased by at least 50%. This change in tendon CSA was used as a proxy to identify the end of the bone-tendon junction region.

In order to assess the validity of using surface markers to measure regional tendon strain, we compared video tracking measurements of surface markers to that of insect pins placed through the tendon. The surface markers and pins were placed in the same locations, with two sets of each spaced approximately 10 mm apart. The comparison of these two techniques were used to reveal potential differences between surface (epitendon) strain and mid-substance strain.

### Statistical Analysis

The influence of treatment group (EXE, RES, and RES-BTX) on animal movement data, tendon morphology and material properties, and bone-tendon junction morphology and material properties was evaluated using one-way ANOVA tests run in R (R Core Team, 2016) and Minitab (Minitab, LLC, State College, PA, United States) when criteria of normality and equal variances were met. *Post-hoc* Tukey tests were run for significant results to determine specific group effects. When criteria of normality and/or equal variance were not met, one-way non-parametric ANOVA on rank (i.e., Kruskal–Wallis) tests were run to determine influence of treatment group, with *post-hoc* Dunn tests (Dunn, 1964). The level of significance was set at α = 0.05 *a priori*. Effect size (E.S.) values were reported as omega-squared analyses, and were computed using the “sjstats” package in R (Lüdecke, 2019).

## Results

### Habitual Movement

Bird movement data did not pass criteria of normality and/or equal variance. Kruskal–Wallis tests indicated no difference between treatment groups in percent time spent walking (*p* = 0.393), percent time spent standing (*p* = 0.312), and percent time spent sitting (*p* = 0.286, adjusted p for ties = 0.241), indicating that these measures were not significantly affected by the treatments (Table 1).

**TABLE 1:** Animal movement analysis.

	EXE	RES	RES-BTX
**Time during growth (%)**			
Standing*	69.2 ± 18.6	66.6 ± 12.4	67.2 ± 15.7
Walking*	26.6 ± 19.3	28.0 ± 12.8	27.2 ± 18.2
Sitting*	3.9 ± 6.5	5.1 ± 5.9	5.3 ± 7.1
**High-intensity movements (counts per day)**			
Sprints*	**347 ± 310**	**64 ± 140**^†^	**103 ± 143**^†^
Jumps*	**210 ± 360**	**0 ± 0**^†^	**0 ± 0**^†^

*Values reported as group means ± standard deviation. Bold type indicates significance with α = 0.05; ^∗^represents non-parametric Kruskal–Wallis testing and *post-hoc* Dunn testing where applicable; ^†^represents values significantly different from EXE group value. Counts per group are interpolated over the 12 lighted hours.*

A Kruskal–Wallis test of number of sprint actions per group per day indicated significant differences between treatment groups (*p* < 0.001). A *post-hoc* Dunn test revealed that EXE birds sprinted significantly more than RES (*p* < 0.001) and RES-BTX birds (*p* < 0.001), but there was no significant difference between RES and RES-BTX birds (*p* = 0.239). EXE birds on average performed approximately 5.5 times more sprint actions than RES birds, and approximately 3.4 times more sprint actions than RES-BTX birds.

A Kruskal–Wallis test of number of jump actions per group per day indicated significant differences between treatment groups (*p* < 0.001). A *post-hoc* Dunn test revealed that EXE birds jumped significantly more than RES (*p* < 0.001) and RES-BTX birds (*p* < 0.001). EXE birds on average performed approximately 210 jump actions per group per day, while no jumps were observed in either RES or RES-BTX birds.

### Tendon Functional Stiffness, Elastic Modulus, and Hysteresis

**FIGURE 5:**
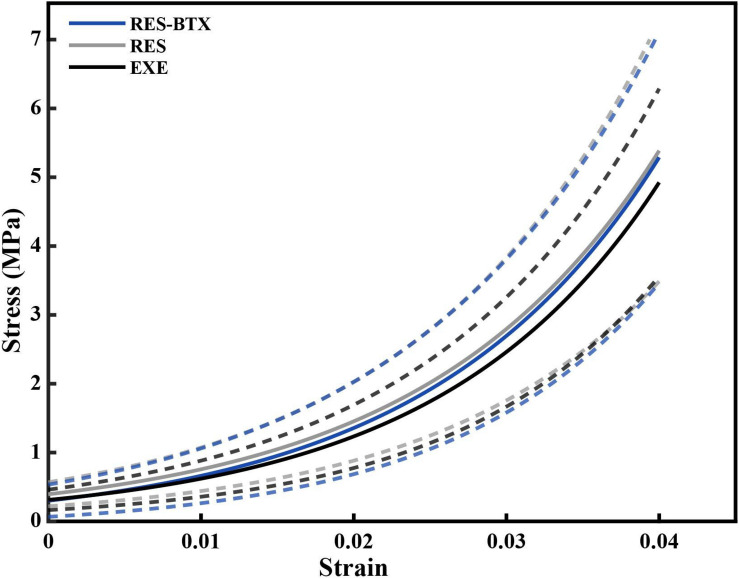
Average stress-strain curve of the loading portion of the load cycle, by condition. Averages were calculated by fitting an exponential curve, *Stress* = *Ae*^B×*Strain*^to each of the last five loading cycles up to 0.04 strain for each tendon. The constants A and B from the fitted exponential equations of the five loading cycles were averaged for each specimen, and used to generate individual curves that could be averaged across the groups (solid lines). Dashed lines indicate one standard deviation above and below the mean for each condition. Gray (RES) and blue (RES-BTX) curves were superimposed and for clarity, RES-BTX curves have been globally offset by 0.1 MPa.

**FIGURE 6:**
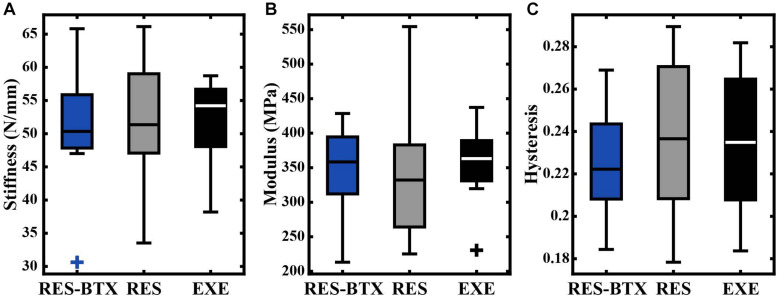
Boxplots of **(A)** stiffness (N/mm), **(B)** modulus (MPa), and **(C)** hysteresis by condition. Crosses (+) indicate values that are outside 1.5 times the interquartile range above the upper quartile and below the lower quartile. No significant differences were found between groups.

**FIGURE 7:**
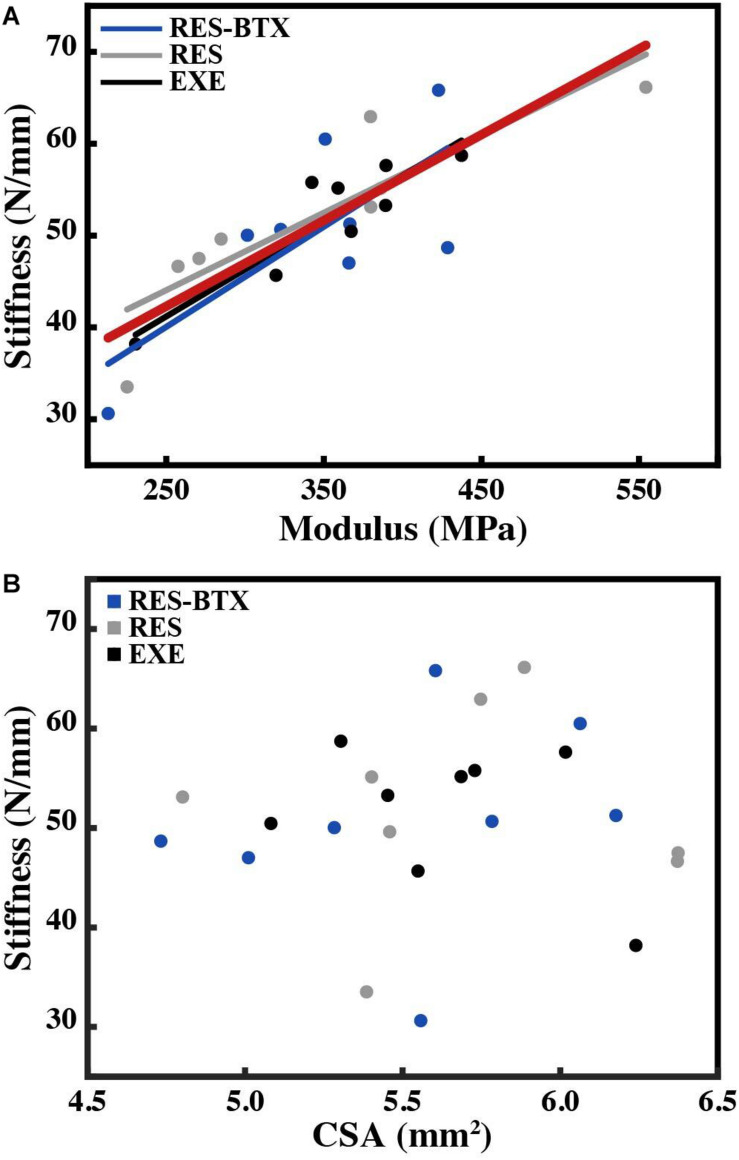
Plot of **(A)** tendon stiffness vs. tendon modulus, with lines of best fit by group and for all animals (red) and **(B)** tendon stiffness vs. average tendon cross-sectional area (CSA, mm^2^). Tendon stiffness across all animals correlates with modulus (*R*^2^ = 0.743, *p* < 0.01), and not with average tendon cross-sectional area (*R*^2^ = 0.007, *p* = 0.698).

All tendon morphology and material property data passed criteria of normality and equal variance, and thus were analyzed using one-way ANOVA testing to determine differences between treatment groups. Body mass (*p* = 0.755, E.S = −0.066) and TMT length (*p* = 0.769, E.S. = −0.068) were not statistically different between groups (Table 2). Neither Achilles tendon length (*p* = 0.79, E.S. = −0.067) nor the average Achilles tendon CSA (*p* = 0.81, E.S. = −0.07) were statistically different between groups. Functional stiffness (*K*_*func*_, *p* = 0.951, E.S. = −0.086), modulus of elasticity (*E*, *p* = 0.955, E.S. = −0.086), and hysteresis (*H*, *p* = 0.760, E.S. = −0.064) were similarly not statistically different between groups (Figures 5, 6). Additionally, tendon stiffness across all animals correlated with modulus (*R*^2^ = 0.743, *p* < 0.01), but not with average tendon cross-sectional area (CSA; mm^2^) (*R*^2^ = 0.007, *p* = 0.698; Figure 7). Hysteresis calculations may have been affected by the fact that not all tendons were pulled to exactly 0.05 strain.

**TABLE 2:** Tendon morphology and material properties.

	EXE	RES	RES-BTX
Body Mass (kg)	1.70 ± 0.14	1.65 ± 0.11	1.70 ± 0.20
Tarsometatarsus Length (mm)	77.7 ± 5.0	77.1 ± 3.2	78.7 ± 4.2
Achilles tendon length (*L*_*0*_, mm)	38.2 ± 1.8	36.8 ± 5.4	37.9 ± 3.9
Average Achilles tendon CSA (mm^2^)	5.63 ± 0.37	5.69 ± 0.53	5.53 ± 0.50
Functional stiffness (*K*_*func*_, N/mm)	51.9 ± 6.92	51.83 ± 10.19	50.57 ± 10.32
Modulus of elasticity (*E*, MPa)	354.4 ± 61.2	342.3 ± 106.3	346.5 ± 69.4
Hysteresis (*H*)	0.24 ± 0.04	0.24 ± 0.04	0.23 ± 0.03

*Values reported as group means ± standard deviation.*

### Regional Tendon Findings

**FIGURE 8:**
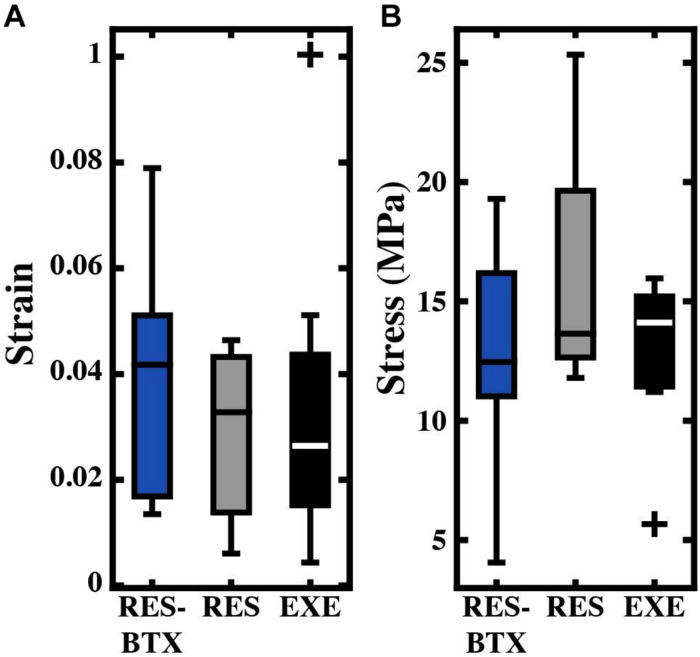
Boxplots of **(A)** strain (mm/mm) and **(B)** stress (MPa) by condition for the bone-tendon junction region. Crosses (+) indicate values that are outside 1.5 times the interquartile range above the upper quartile and below the lower quartile. Values used were taken when the total tendon strain equaled 0.04. No significant differences were found between groups.

Regional strain, minimum CSA, and tendon stress at the bone tendon-junction at 0.04 global strain did not pass criteria of normality and/or equal variance, and thus were analyzed using the Kruskal–Wallis tests (Table 3). Regional strain within each tendon specimen varied by up to 15% (Figure 5). Regional strain experienced by the thinnest portion of each sample (i.e., the bone-tendon junction region) when global strain was equal to 0.04 were not statistically different between groups (*p* = 0.608, E.S. = −0.057; Figure 8). CSA for the thinnest portion of each sample was 2.8% greater for RES animals and 13.7% smaller for RES-BTX animals when compared to EXE animals, but this was not a statistically significant difference (*p* = 0.099, E.S. = 0.115). Tendon stress at the bone tendon-junction at 0.04 global strain was 4.65% less for RES-BTX animals and 25.13% greater for RES animals when compared to EXE animals, but neither was this difference statistically significant (*p* = 0.570, E.S. = 0.025; Figure 8).

**TABLE 3:** Bone-tendon junction morphology and material properties.

	EXE	RES	RES-BTX
Strain at BTJ*	0.03 ± 0.03	0.03 ± 0.02	0.04 ± 0.02
Minimum Achilles tendon CSA (mm^2^)*	2.48 ± 0.48	2.14 ± 0.38	2.55 ± 0.29
Stress at BTJ (N/mm^2^)*	12.89 ± 3.38	16.13 ± 5.60	12.83 ± 4.74

Values reported as group mean ± standard deviation. * Represents non-parametric Kruskal–Wallis testing and *post-hoc* Dunn testing where applicable.

Our surface-marker to pin-marker test found that the two techniques are comparable. The techniques showed an average difference in maximal strain of the tendon region of 0.00110 ± 0.00025. This small difference equates to an approximately 5% difference between the techniques, indicating that the surface marker strain is similar to mid-substance strain.

## Discussion

The goal of this study was to examine developmental plasticity of growing tendon. Contrary to our hypotheses, the spring-like characteristics of the free tendon and the bone-tendon junction were unaffected both after restricting high-intensity exercise and after restricting high-intensity exercise and additional chronic administration of botulinum toxin across the growth period. These results suggest that tendon properties, at least in the bird species studied here, may be resilient to variations in load level during the growth period.

### Tendon Response to Altered Growth Conditions

Stiffness, modulus and cross sectional area values of the free tendon in all groups are very close to the values reported for comparable control animals in a previous study of adult guinea fowl (stiffness: 51.9 ± 6.92 EXE from our study vs. 50.58 ± 10.62 N/mm, modulus: 354.4 ± 61.2 EXE from our study vs. 338 ± 11 MPa, CSA: 5.63 ± 0.37 EXE from our study vs. 6.45 ± 0.30 mm^2^; Buchanan and Marsh, 2001). These values are similar, despite the differences between the *in vivo* experimental techniques used to test tendon mechanics by Buchanan and Marsh (2001) and the explanted material testing methods adopted here.

Unlike the marked increase in stiffness and modulus resulting from running exercise in adult animals (Buchanan and Marsh, 2001), we did not observe tendon adaptation as a result of restricting movement or BTX-A administration in growing animals. While very little difference in tendon properties was observed between groups, there was variation in tendon stiffness within each group (Figure 5). In each group this variation is accounted for primarily by tendon modulus rather than tendon CSA, further indicating that the mechanical characteristics of the tendon remained similar across groups.

The similarity of tendon properties across groups is surprising considering the difference in activity level of exercised birds which engaged in substantially more daily high-intensity movements (jumping, running) than the restricted birds. These high-intensity movements are known to generate considerably higher tendon loading than those observed during walking and standing. In particular, landing from perches is expected to result in considerable eccentric muscle force. Indeed, on a daily basis, the EXE group performed approximately 210 perch jumps/landings and 347 running maneuvers. In contrast, jumping and landing was completely absent in the RES and RES-BTX animals and running maneuvers were considerably lower. It is difficult to know how this load reduction treatment compares to that applied in the previous growth study of domestic fowl by Nakagaki et al. (2007). These authors did not report specific activity levels of their groups, but attributed lower tendon stiffness in caged animals (compared to pen-reared animals) to the inability of these animals to engage in spontaneous running exercise. The change in tendon load stimulus between the EXE and both the RES and RES-BTX groups is arguably greater than the twice-weekly resistance training intervention shown to induce Achilles tendon adaptation over a 10-week period in children (Waugh et al., 2014). Comparing our study to short-term exercise interventions is nevertheless difficult for several reasons. First, these studies are increasing as opposed to decreasing a baseline tendon load. Secondly, while they are conducted during development, these studies comprise a very small portion of the individuals’ growth span, and therefore do not capture the prominent remodeling of tendon associated with growth (Curwin et al., 1994; Wren et al., 1997; Waugh et al., 2012; Mersmann et al., 2017b).

The lack of tendon plasticity in the RES-BTX group is also surprising considering muscle paralysis and tendon unloading is typically expected after botulinum toxin treatment (Longino et al., 2005; Schwartz et al., 2013). Unlike previous investigators, we administered BTX-A bilaterally, preventing single-leg unweighting known to occur in some quadrupedal rodent model studies (Longino et al., 2005; Fortuna et al., 2015). It is possible, therefore, that by maintaining their bipedal stance and gait the guinea fowl in this study maintained loading of their gastrocnemius muscles to some degree. Alternatively, passive muscle force might have been developed if the gastrocnemius muscles were maintained at longer lengths. Either of these scenarios may have mitigated the stress shielding we had expected from our BTX-A treatment. After BTX-A injections, we observed qualitatively a short-term (1–2 days) reduction in standing and an affected gait (especially in younger animals). However, posture and gait quickly returned to normal, consistent with the notion of a muted BTX-A effect. It is also important to note that other analyses of chronic BTX-A treatment in growing animals have also resulted in minimal changes to tendon properties (Eliasson et al., 2007). This may indicate a different BTX-A response in rapidly growing animals compared to mature animals or human children (e.g., cerebral palsy treatment) who have slower growth rates.

### What Can Explain the Lack of Tendon Plasticity Across the EXE, RES, and RES-BTX Groups?

**FIGURE 9:**
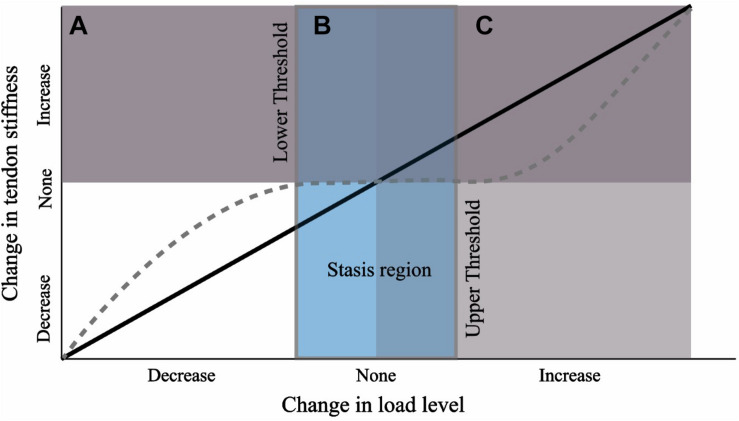
Hypothesis for tendon response to a change in load level during growth from a nominal baseline value. Black line depicts a linear relationship. We propose the response curve may more closely resemble the dashed gray curve where a threshold of change in load must be crossed before triggering a plastic response. The wide stasis region may result due to a strong load-independent biological growth component (e.g., biological growth factors).

We suggest that the results of this study can best be explained if we reject the hypothesis that there is a constant load-dependent relationship between the external load level and tendon stiffness during growth. Instead, our results are consistent with the theory that there are lower and upper thresholds of external load stimulus necessary to induce a plastic response (Figure 9). It has been suggested that a threshold of tendon connective tissue strain exists below which a catabolic response is initiated (Brown et al., 1998; Lavagnino and Arnoczky, 2005; Arampatzis et al., 2007). *In vitro* studies have shown that this lower homeostatic strain limit can be as little as 0.025 (Nirmalanandhan et al., 2008) or 0.01 if the appropriate strain frequency is applied (Lavagnino et al., 2003). Similarly, protection against tendon degradation due to immobilization, including a reduction in modulus, has been shown to occur *in vitro* if loads as low as 1 MPa are maintained (Hannafin et al., 1995; Arnoczky et al., 2003). In our study, all groups had an equal amount of low-intensity exercise, but standing and walking comprised the majority of the daily activity (Table 1). Surprisingly, even BTX-A administration did not alter the amount of time spent standing and walking, and for reasons explained above, BTX-A animals might have loaded their tendons during these activities. We posit this frequent low-intensity loading could surpass the relatively low stress and strain thresholds that have been proposed for inhibiting tendon degradation (low threshold in Figure 9). Although we do not have *in vivo* experimental values for tendon stress or strain, or the relative intensity of the muscle-tendon load, muscle data provided in Cox et al. (2019) allow for simple estimates of these parameters. Using the maximum isometric force (*F*_max_) of the gastrocnemius muscles (scaled to body mass) and their pennation angles, we can predict a theoretical maximal force in the Achilles tendon. The level of activation (*A*, scaled from 0 to 100%) required to generate 1 MPa tendon stress can subsequently be predicted from a simple linear activation-force relationship and the tendon *CSA*_*avg*_ from the experimental animals:

$$A=\frac{1\,\text{MPa}\cdot C\,S\,A_{\text{avg}}}{F_{\max}}$$

where the *CSA*_*avg*_ is taken as the average value from the three experimental groups. This prediction yields an activation level of 4% required by the gastrocnemius muscles to generate 1 Mpa stress in the Achilles tendon. This equation does not take into account force reductions due to dynamic force-length and force-velocity affects. If we assume these losses were as high as 50%, we predict an activation level of 8%. Even this upward adjustment in activation level is likely achieved in standing, and surpassed in walking, based on experimental muscle activity and blood flow measurements (Daley and Biewener, 2003; Marsh et al., 2004). Thus, if the lower threshold for tendon maintenance is low, as *in vitro* data indicate, the animals in this study may have readily met this threshold. In fact, an experimental *in vivo* activation level of 38% has been reported for walking guineafowl (Daley and Biewener, 2003). Furthermore, linking the *in vivo* forces measured during walking in this study with the reported tendon CSA yields a stress of 4.6 MPa in the lateral gastrocnemius tendon (Daley and Biewener, 2003). Thus, the stress in the Achilles tendon may have well exceeded 1 MPa during much of the daily activity in this study. It is also notable that a stress of 1 MPa in the tendons analyzed in this study will generate strains in excess of 0.01 (Figure 4).

An unexpected conclusion arising from this study, therefore, is that tendon appears to continue to develop and is resilient to altered external load levels so long as a minimum threshold of tendon load is maintained. A similar view of load-resilient tendon growth was proposed by Eliasson et al. (2007) in their study of Achilles tendon response to chronic BTX-A treatment in growing rats. These authors likewise found no changes in Achilles stiffness and strength and proposed that during the growth period, tendon size and strength are under systemic control and that there is a lower-bound threshold of tendon loading that, if surpassed, permits tendon maintenance.

A baseline of low-intensity activity might also help explain the discrepancy between our results and those of Nakagaki et al. (2007), who reported a reduction in Achilles tendon stiffness in their activity-restricted domestic fowl. Guinea fowl are game birds that, unlike domestic fowl, exhibit high activity levels even when space is restricted. Similar to other wild species of fowl (e.g., junglefowl; Dawkins, 1989), the percentage of time spent resting was very low in all groups and they were recorded walking approximately 25% of the observed time (Table 1). A rough estimate of the daily distance walked in our study (based on time spent walking and estimated speed of 0.5 m/s) is approximately 5.9 km per bird. In comparison, the time spent at rest (sitting) is approximately 60–70% in domestic fowl, with as little as 3–10% of time spent walking (Hansen, 1994; Cornetto and Estevez, 2001; Bizeray et al., 2002). Even in free-range housing, domestic fowl spend approximately 50% of the time at rest (Rodriguez-Aurrekoetxea et al., 2015). If the caged birds from the study of Nakagaki et al. (2007) were (presumably) more sedentary than our animals, they may not have reached a lower tendon load threshold required for tendon maintenance. As Woo et al. (1982) observed, extreme disuse has the potential to initiate a rapid decline in tendon properties. From this perspective, their findings might demonstrate a disuse response as opposed to changes in the tendon induced by high loads.

A potentially strong contributing factor to our proposed “mechanical threshold” hypothesis is non-mechanical mediated growth. The lack of tendon adaptation between EXE and the other groups may arise because the load-independent biological component of tendon development overwhelmed the mechanobiological component (mechanically stimulated development; Wren et al., 1997). Tendon development has been shown to be under the control of several cellular and extracellular matrix signal pathways and growth factors (for reviews see Birk and Zycband, 1994; Gaut and Duprez, 2016). These biological components play a part in the marked alteration in tendon properties across the growth period; for example, as has been pointed out previously, a 40,000-fold increase in elastic modulus occurs between chick fetal tendon and adult tendon (Gaut and Duprez, 2016), and a 30-fold increase in chick tendon tensile strength has been reported over only a 2 week post-natal development period (Silver et al., 2003). If the tendon has a strong load-independent biological growth component, this might outweigh the mechanobiological response arising from altered exercise loads. Furthermore, growth-mediated mechanotransduction arising from changes in body weight may likewise dominate any remodeling signal arising from variation in external load (e.g., movement). Together, these growth-mediated factors may explain why the lower and upper threshold of external exercise load required for tendon remodeling in a growing animal maybe difficult to penetrate, resulting in a wide “stasis region” (Figure 9). This interpretation is consistent with another study that implemented long-term exercise training in growing horses (Kasashima et al., 2008). In that study, a baseline level of low-intensity activity appears to have been retained across the experimental and control groups (i.e., lower threshold for tendon maintenance is met) and in which the tendons of interest did not exhibit stiffness adaptations (i.e., upper threshold for tendon remodeling is not met).

A second possible explanation for the lack of tendon response in the EXE group is if the mechanical characteristics of the altered tendon load required for tissue adaptation were not met by our treatment. It is generally understood that strain-mediated mechanotransduction regulates tendon homeostasis (Wren et al., 2000; Arnoczky et al., 2002; Arampatzis et al., 2007). However, the characteristics of the applied strain, including the strain magnitude, strain duration and repetitive strain rate influence tendon adaptation (Arnoczky et al., 2002; Yamamoto et al., 2005; Arampatzis et al., 2007; Bohm et al., 2014). Studies of increased loading on intact human Achilles tendon indicate that the most pronounced tendon remodeling occurs under both large strain magnitudes and long strains durations, with a lack of adaptation at low strains (Arampatzis et al., 2007, 2010). A similar reliance on strain magnitude for tendon adaptation has been observed in *in vitro* studies of both tendon load depravation and load augmentation (Arnoczky et al., 2002; Nirmalanandhan et al., 2008; Wang et al., 2013, 2015). The high-intensity activities undertaken by the EXE group, in particular jumping and landing, generate large Achilles forces and strains in guinea fowl and other ground birds (Henry et al., 2005; Konow et al., 2012; Arellano et al., 2019). The duration of the strain application is also long in these movements, allowing for tendon pre-stretch in jumping and energy damping during landing (Roberts and Azizi, 2011; Roberts, 2016). This suggests that the removal of these high-intensity activities in the RES and RES-BTX groups may have been well suited for inducing tendon adaptation (i.e., reduced cross-sectional area, stiffness, modulus or hysteresis). It is also plausible that it is only an increase in strain and strain duration that leads to tendon adaptation, whereas a large reduction in strain and strain duration from normal habitual movements (predicted in the RES and RES-BTX groups in this study) does not. Furthermore, data from human training studies indicate that repetitive loading may be required for Achilles tendon adaptation (Bohm et al., 2014). It remains possible that removal of repetitive loading, in particular, is also required to induce an unloading response in the tendon. The high-intensity activities in the EXE occurred intermittently, possibly minimizing the effectiveness of removing these loads in the RES and RES-BTX groups for generating an adaptive stimulus.

### Functional Implications of Unaltered Tendon Mechanical Properties

The Achilles tendon is essential for elastic energy storage and return, as well as for amplifying power production in acceleration movements and assisting energy dissipation during landing (Roberts and Azizi, 2011; Roberts, 2016). Our data indicate that these functions are likely unaltered between our experimental groups, although how the tendon interacts with other possible modifications in other tissues, for example muscle, is not known. Our previous companion study on the same EXE and RES animals found that the allowance of high-intensity movements over the growth period resulted in greater maximum center of mass vertical jump velocity and peak jump force, work, and power once the animals reached adult age (Cox et al., 2020). Despite functional differences, the amount of extensor muscle mass, including that of the gastrocnemius muscles, was not different between groups. This led to the conclusion that neural factors, rather than muscle adaptation, might be more closely associated with the improved performance in the EXE group. Our data showing unaltered Achilles tendon properties between the EXE and RES group further supports this conclusion.

Unlike the studies of Eliasson et al. (2007) and Khayyeri et al. (2017), we did not observe any effect on tendon hysteresis. This finding implies that, not only is the capacity for tendon elastic energy storage likely unaltered (if tendon stiffness is unaltered), but that the amount of energy returned in cyclical loading is likely also unaffected by loading history during growth. These data help toward a mechanistic explanation for the similar energy cost of steady-state running between EXE and RES birds reported in Cox et al. (2020). Energy recycling in tendon, in particular the Achilles tendon, has been proposed as a major factor determining locomotor energetics, including in running birds and other animals (Biewener and Baudinette, 1995; Roberts et al., 1997; Lichtwark and Wilson, 2008; Roberts, 2016). It follows that if this property is unaffected during growth that adult running economy may also remain unchanged. It is, however, of note that the hysteresis values recorded in this study are relatively high compared to many other tendons reported in the literature [see for example Bennett et al. (1986) and the summary by Finni et al. (2012)]. High hysteresis values may exacerbate tendon hyperthermia (Wilson and Goodship, 1994). Other factors, such as heat dissipation and cell heat tolerance (Birch et al., 1997) may also need to be considered to understand whether the high hysteresis affects tendon damage in guineafowl. Also, while the classic view is that tendon has a hysteresis of less than 10%, there are several examples of tendon having higher hysteresis values in both humans (Finni et al., 2012) and other species (Shadwick, 1990; Pollock and Shadwick, 1994 (see select species); Vereecke and Channon, 2013). Finally, we cannot rule out the possibility that our methodological approach resulted in a higher hysteresis than what occurs *in vivo*. For example, it is possible that if the entire tendon-aponeurosis complex was tested, or if a more realistic strain rate or loading duration was used, that we would observe lower hysteresis values.

Our results also suggest that tendon strength may have been unaltered across groups. The bone-tendon junction has previously been identified as the point of failure in other studies of tendon mechanics (Jack, 1950; Woo et al., 1983; Woo and Buckwalter, 1988; Jarvinen, 1992; Wren et al., 2001, 2003). Consistent with these studies, the region at the Achilles bone-tendon junction in the guinea fowl has the smallest cross-sectional area and is thus susceptible to the highest stress. The absence of changes to the material or geometric properties at the bone tendon junction suggests tendon strength may have been preserved across groups (experimental failure mechanics were not investigated in this study). The safety factor of the tendon may therefore be relatively higher in the RES and RES-BTX groups that do not engage in high-intensity movements. Interestingly, a lack of modulation in tendon safety factor indicates less economical use of tissue in the RES and RES-BTX groups (Alexander, 1981; Gosline, 1992), but supports the hypothesis that tendon growth is under systemic control (Eliasson et al., 2007).

Finally, our data have implications for both broader evolutionary aspects of musculoskeletal plasticity and for human musculoskeletal health. A tendon relatively insensitive to growth-period loading history may present a selective advantage, as sudden, unpredictable changes in environment will not change the tendon’s mechanical and functional growth trajectory. This may be especially effective in fast growing species, such as birds. The evolutionary adaptive consequences of tendon plasticity are nevertheless complex and other non-adaptive scenarios may also be applicable. Our data could also be taken as a sign that maintaining a minimum load threshold could be an important goal for preserving proper tendon growth and health. For example, the lack of tendon adaptation seen here indicate a lower sensitivity to disuse compared to muscle, and may help guide studies addressing the relative responsiveness of these tissues to disuse and movement function (Mersmann et al., 2017b). However, we caution against making direct inferences to human development, and rather see our data as hypothesis-generative.

### Limitations

The results of this study suggest that, contrary to the intention of our experimental design, Botulinum toxin may not have induced considerable further tendon unloading beyond that due to restricted movement alone. We observed qualitative changes in the animals’ posture and time spent standing in the 2 days immediately post injection when animals were housed in cages for observation, but we did not record specific movement behavior or mechanics. These deficiencies, however, rapidly diminished and movement returned to normal when the animals were returned to pen housing as indicated by our movement scores. To test the efficacy of Botulinum toxin in adults of this species, we also performed BTX-A injections in a group of adult animals that were part of a separate study. In these animals we observed a marked reduction in movement ability, including a loss of jumping behavior. Detailed measurements of the physiological effect of BTX-A-induced paralysis were, nevertheless, not performed in the present study. For example, we do not have electromyography recordings, reflex tests or data on the amount of force reduction in the tendon post BTX-A injections that could validate the BTX-A treatment. As discussed previously, it therefore remains possible that over the course of the growth period the average tendon loading was not greatly reduced below that of the RES group.

This study measured the material properties of the free tendon, but this may not fully capture variations in elasticity in this muscle-tendon unit. These properties matched very closely the properties measured by Buchanan and Marsh (2001), despite their use of a different *in vivo* technique that incorporated the free tendon and aponeurosis. Nevertheless, aponeurosis strain has been shown to be an important component of tendon strain (Roberts et al., 1997; Arellano et al., 2019) and dynamic changes in aponeurosis strain has been shown to modulate overall tendon stiffness *in vivo* (Azizi and Roberts, 2009; Arellano et al., 2019). Further analyses incorporating material properties of the aponeurosis might reveal load-induced plasticity over the growth period different to those reported here. We also did not experimentally test tendons to failure. While our measurements of the bone-tendon junction provide indirect information relevant to tendon strength, experimental measurements of tendon failure might reveal differences not observed in the present study.

Findings from this study are based on linking average group movement and tendon material data. Our methods did not permit us to link an individual bird’s movement data to its corresponding tendon material properties. It is likely that activity levels varied between animals, and that this contributed to variation in tendon properties that we could not account for. However, as our previous study (Cox et al., 2020) found, the marked restriction in high-intensity activities resulted in a reduction in jumping performance in the same animals as in this study, indicating that the treatment did alter locomotor function, but not the properties of the Achilles tendon as determined from our group average comparisons.

Finally, we caution against making direct comparisons to human tendon growth and plasticity. The guinea fowl provide a valuable model for tendon loading and growth. In particular, their bipedal gait result in limb loading characteristics that have many similarities to humans (Rubenson and Marsh, 2009; Rubenson et al., 2010). This overcomes some of the limitations of using quadrupedal rodent models in musculoskeletal research (Hu et al., 2017). There are other physiological characteristics of our fowl model that might affect comparisons to humans; in particular, their very rapid growth might result in a different tendon response to altered load compared to that of the relatively slower postnatal development of humans.

Here we aimed to help resolve the effect of unloading on tendon by implementing a scope of altered tendon load across the entire growth span in an avian bipedal model. In conclusion, we found the growing Achilles tendon in guinea fowl insensitive to variations in disuse stimuli. A lack of change in stiffness or modulus suggest thresholds of load variation exists that must be surpassed to induce mechanical adaptation to the growing tendon.

## Data Availability Statement

The data are available from the Penn State University data repository site, ScholarSphere https://doi.org/10.26207/v5gr-7e21.

## Ethics Statement

The animal study was reviewed and approved by The Pennsylvania State University Institutional Animal Care and Use Committee.

## Author Contributions

KK, SC, MS, TR, SP, and JR contributed to the conception and design of the study. KK, SC, MS, AD, MH, TN, and JR developed the methodologies and collected the data. KK, SC, MS, and JR analyzed the data and contributed to figure preparation. KK and JR drafted the initial manuscript. All authors contributed to the manuscript drafting and editing. All authors contributed critically to the data interpretation and approved the final manuscript.

## Conflict of Interest

The authors declare that the research was conducted in the absence of any commercial or financial relationships that could be construed as a potential conflict of interest.
